# The benefits of skull stripping in the normalization of clinical fMRI data^☆^

**DOI:** 10.1016/j.nicl.2013.09.007

**Published:** 2013-09-30

**Authors:** F.Ph.S. Fischmeister, I. Höllinger, N. Klinger, A. Geissler, M.C. Wurnig, E. Matt, J. Rath, S.D. Robinson, S. Trattnig, R. Beisteiner

**Affiliations:** a Study Group Clinical fMRI, Department of Neurology, Medical University of Vienna, Austria; b High Field MR Center, Medical University of Vienna, Austria; c Department of Biomedical Imaging and Image-guided Therapy, Medical University of Vienna, Austria

**Keywords:** Skull-stripping, Normalization, Lesion, Functional MRI, Clinical brain mapping, Patients

## Abstract

Establishing a reliable correspondence between lesioned brains and a template is challenging using current normalization techniques. The optimum procedure has not been conclusively established, and a critical dichotomy is whether to use input data sets which contain skull signal, or whether skull signal should be removed. Here we provide a first investigation into whether clinical fMRI benefits from skull stripping, based on data from a presurgical language localization task. Brain activation changes related to deskulled/not-deskulled input data are determined in the context of very recently developed (New Segment, Unified Segmentation) and standard normalization approaches. Analysis of structural and functional data demonstrates that skull stripping improves language localization in MNI space — particularly when used in combination with the New Segment normalization technique.

## Highlights

• First investigation of the possible effects of skull-stripping with clinical fMRI data.
• Comparison of standard and most recent normalization approaches.
• Skull stripping improves language localization in MNI space.

## 1 Introduction

Precise and valid spatial normalization into a common space across all subjects is one of the key components in group analysis of structural and functional neuroimaging data (Brett et al., 2002). In recent years a wealth of algorithms and methods have been developed to account for and correct inter-subject variability in healthy subjects' brains (for a recent review and comparison of algorithms see Klein et al., 2009, 2010). Most normalization methods use automated algorithms to minimize the difference between a subjects' image and a standardized template by applying linear and nonlinear transforms. The establishment of a reliable and robust correspondence between subjects' brains and a template is difficult, however, when there are inherent contrast differences between the two. Disparate B_0_ signal dropout, B_1_ inhomogeneity and differing tissue contrast can arise from acquisition at different field strengths or from the use of different measurement parameters. The situation becomes particularly problematic in the normalization of lesioned brains, since focal brain lesions or loss of brain tissue resulting from stroke, tumors, or surgery may lead to a lack of correspondence between patient images and standardized templates due to biased normalizations or overfitting (Brett et al., 2001). The impact of such a lack of correspondence in patients' brains to templates on the analysis of functional imaging data has been highlighted in a large body of work (Beisteiner et al., 2010; Crinion et al., 2007; Gartus et al., 2007; Hoeksma et al., 2005; Tahmasebi et al., 2009; Vandenbroucke et al., 2004; Yassa and Stark, 2009). Most clinical studies apply normalization techniques implemented in SPM and, until recently, the SPM standard normalization approach was most popular. However, the Unified Segmentation Model approach (Ashburner and Friston, 2005) constitutes a significant advance in normalization quality. Unified Segmentation attempts to capture all aspects of an anatomical image using a probabilistic framework with tissue prior maps (TPMs) and thus enables tissue classification, bias correction due to signal inhomogeneities, and nonlinear image registration in one model. Crinion et al. (2007) demonstrated that Unified Segmentation produces significantly better and more reliable anatomical co-localization than any of the conventional normalization approaches which employ cost-function masking (CFM) to deal with pathologies (Brett et al., 2001). More recently, Andersen et al. (2010) showed that for larger lesions resulting, for example, from strokes, the benefit of the Unified Segmentation Model can be further increased when used in addition to CFM rather than instead of it. The most recent development is the New Segment toolbox (SPM manual, FIL Group), introduced into SPM as a “work in progress” package. It utilizes the Unified Segmentation algorithm with an improved registration model and an extended set of tissue probability maps.

A critical factor not well investigated is the influence of skull-stripping or scalp editing to remove non-brain areas before normalizing brains, although this is relevant to all the normalization techniques. Skull stripping may improve the robustness of the registration process, since high resolution structural images contain considerable amounts of non-brain tissue such as eyeballs, bone, skin, and other tissues while the template images either do not, or only do to a certain extent. For voxel based morphometry (VBM) Fein et al. (2006) and Acosta-Cabronero et al. (2008) have already demonstrated that misregistrations of individual brains to a common template could be reduced by using brain-extracted images as initial input data sets. Despite these results, no investigations to date have examined the possible benefits of skull stripping as a postprocessing tool for clinical fMRI. Here, we provide the first detailed structural and functional investigation into whether or not skull-stripping (in the context of 3 different normalization approaches) influences the localization of brain function in a cohort of pathological brains which is typical for clinical functional diagnostics.

## 2 Materials and methods

### 2.1 Patients and paradigm

Patients referred for functional localization of language-related areas as part of presurgical evaluation were selected from a pool of data acquired on a 3 Tesla TIM Trio system (Siemens, Erlangen, Germany) according to the following criteria: (1) localization of the tumor, lesion or epileptic focus within the left hemisphere in the vicinity of the Broca or Wernicke area without any previous surgical excision, (2) the patients were right handed and older than 18 years of age, (3) patients were in a good general state of health with no unrelated clinical symptoms and good cooperation at the time of measurement and (4) there was unequivocal left hemispheric language dominance according to the local clinical fMRI report generated on individual non-normalized fMRI data (Foki et al., 2008), which served as functional gold standard in this study.

**Fig. 1:**
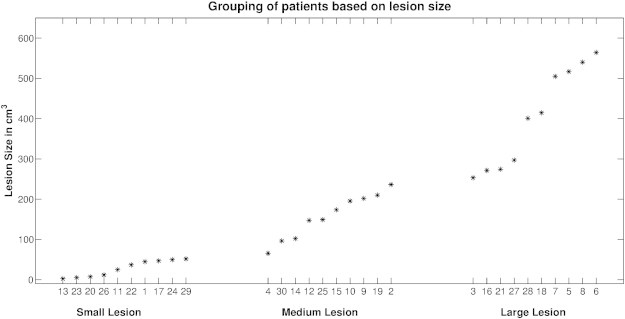
Histogram of lesion size across the three lesioned brain groups. The numbers on the abscissa correspond to the patient numbers listed in Table 1.

36 patients (22 male, 14 female, mean age 42.5 years) fulfilling the above criteria were included in this study (see Table 1). These patients and four healthy subjects (2 male, 2 female, mean age 33.75 years) were subdivided into four equally sized groups according to the extent of the lesion (calculated from the lesion mask). These groups were no-lesion (comprising healthy subjects and epileptic patients), small-lesion, medium-lesion and large-lesion (see Fig. 1). These subgroups were formed to assess the effects of lesion size on normalization differences related to skull-stripping.

Participants performed a simple overt language paradigm developed for a comprehensive test of all language components (Foki et al., 2008; Gartus et al., 2009). It consisted of 20 runs, each lasting 140 s. Each run comprised 3 active blocks alternating with 4 rest blocks, with each block lasting for 20 s. During the active phases, two German sentences were presented to the participants visually (for 10 s each). These sentences consisted of 4 words – the stem of a sentence – presented word by word, followed by two verbs displayed one above the other, constituting a correct and an incorrect possible ending of the sentence. The incorrect verbs were either grammatically wrong or semantically unsuitable. While reading the sentence out loud, subjects were explicitly required to choose the word which forms a correct German sentence.

The study was approved by the ethics committee of the Medical University of Vienna. All patients gave written informed consent.

### 2.2 fMRI acquisition

Images were acquired with a 3 Tesla TIM Trio system (Siemens, Erlangen, Germany) using a 32 channel head RF coil and a head fixation helmet (Edward et al., 2000). Functional MRI data were acquired using single-shot gradient-recalled EPI with 34 axial slices (1.8 × 1.8 mm in-plane resolution, 3 mm slice thickness, matrix size of 128 × 128, a FOV of 230 mm, echo time (TE) 35 ms, repetition time (TR) 2500 ms and GRAPPA acceleration factor 2), aligned to the anterior and posterior commissures. Two dummy/preparation scans were prefaced each run to ensure quasi-equilibrium in longitudinal magnetization. High-resolution T1-weighted MR images were acquired using a 3D MPRAGE sequence (TE = 3.02 ms, TR = 2190 ms, inversion time (TI) = 1300 ms) with a matrix size of 250 × 250 × 256, with isometric voxels with a nominal side length of 0.9 mm, flip angle of 9° and a GRAPPA acceleration factor of 2.

### 2.3 Image preprocessing

First, binary masks delineating lesions in original unprocessed anatomical T1 images were defined manually in the native space of each patient using MRIcron (Rorden and Brett, 2000). Although it has been repeatedly shown that the quality of the mask has limited influence on the normalization results, tumor boundaries were outlined as precisely as possible by experienced clinical fMRI experts (FF, RB) (Andersen et al., 2010; Brett et al., 2001). Lesion masks generated in this way were smoothed with an 8 mm FWHM Gaussian filter as recommended by Brett et al. (2001) and constrained so as not to extend beyond the brain.

In a separate step, brain extracted images, i.e. the deskulled anatomical images, were obtained using FSL's (Software library of the Oxford Centre for Functional MRI of the Brain (FMRIB): http://www.fmrib.ox.ac.uk/fsl/) brain extraction tool (BET2; Smith, 2002) followed by manual removal of residual non-brain areas, again using MRIcron. To this end, a mask was drawn capturing residual non-brain areas including bone, fat, and meninges and added to the brain mask resulting from BET2. The amount of manual editing needed was comparable for the four lesion groups. This combined mask was applied to individual T1 scans, resulting in clean deskulled anatomical images.

Image processing, involving the different normalization pipelines, preprocessing and statistical analysis of the functional data was performed using SPM8 (Software library by the members & collaborators of the Wellcome Trust Centre for Neuroimaging (Functional Imaging Laboratory Group); http://fil.ion.ulc.ac.uk/spm) and largely followed the steps described by Crinion et al. (2007). Default parameters were chosen for all analysis steps – except where noted in the following description – to keep the normalization and analysis procedures as close as possible to that used in current practice. Normalization of the structural and functional images involved two steps. Step I: generation of a common spatial starting point; ensuring that images had the same rotation and origin as the MNI template by applying an affine 3D rigid-body transformation. Step II: standard SPM normalization (Ashburner and Friston, 1999), Unified Segmentation normalization (Ashburner and Friston, 2005) and New Segment normalization (SPM manual, FIL Group) using skulled and deskulled input data sets.

#### 2.3.1 Step I (see Fig. 2)

The estimation of different parameter sets to transform the data to MNI space. First, to account for residual small-scale motion, motion correction parameters were calculated for the functional images using the individual mean EPI image as the reference image. To minimize interpolation errors, these parameters were calculated but not applied to the individual images at this stage, i.e. images were not resliced or resampled in this step. Secondly, both deskulled and not-deskulled structural T1 images were transformed to the individual mean EPI image, calculating EPI-space transformation parameters and then transforming to MNI space using affine 3D rigid-body transformation with the standard SPM T1 template as reference. MNI transformation parameters were thereby generated. These two parameter sets (motion correction parameters and MNI transformation parameters) were then combined to generate a combined transformation which was applied to the functional EPI data. The same procedure was applied to the structural and lesion mask images by combining EPI-space and MNI transformation parameters. Although this is not usually required at this stage of the data analysis, all data sets were resliced then resampled to 2 × 2 × 2 mm voxel size for the functional data and 1 × 1 × 1 for the anatomical data. This step resulted in a common starting point for the subsequent normalization pipelines and was conducted to exclude any confounding effects. Among these are possible distortions resulting from prior non-applied transformations, e.g. Unified Segmentation required the images to be in the approximate position of the MNI space before starting the normalization while standard normalization does not.

To check for possible differences between skulled and deskulled images introduced by the linear transformations of Step I, we performed two analyses. (1) Comparison of skulled with deskulled T1 images after registration of T1 to the mean EPI. (2) Comparison of skulled with deskulled T1 images after Step I had been completed (i.e. after generation of a uniform starting-point for all 6 normalizations). This was done by calculating DICE similarity indices (Dice, 1945) for the skulled/deskulled T1 images. These provide a direct measure of the structural differences between skulled and deskulled T1 at stages (1) and (2). DICE calculations were performed separately for the 4 different lesion groups and with the approach described below (section “Evaluation of structural differences between normalized and template images”). The comparison of skulled with deskulled T1 images was carried out with the deskulled image serving as the reference and the skulled image as the template.

#### 2.3.2 Step II (see Fig. 3)

The default parameters implemented in SPM8 were used for the standard normalization scheme, except for the specification of the template image. Since the MNI152 template provided by SPM8 contains scalp, skull, and meninges, the brain extracted and the standard MNI152 templates provided by FSL were used as references (Fein et al., 2006). For consistency with the SPM template, both FSL templates were smoothed using an 8 mm FWHM Gaussian kernel and then used as reference images to normalize the stripped and non-stripped individual brains. For the Unified Segmentation Model, all parameters including the probabilistic prior maps were left unchanged (following Andersen et al., 2010; Crinion et al., 2007). In accordance with these studies, the number of Gaussians for the “other prior map” (see above) was left unchanged, i.e. it was assumed that the number of different intensity distributions within this tissue map would not be changed by stripping off the skull. Both approaches were conducted with cost-function masking to weight brain lesions appropriately (Andersen et al., 2010) for the three lesioned brain groups. The New Segment algorithm does not support cost function masking but is designed to ignore voxels with a value of zero, which is essentially identical to a cost-function masking approach (personal communication with John Ashburner, FIL methods group). Therefore, lesioned neuronal areas within the anatomical images were first discarded by setting their voxel-values to zero and the resulting “cleaned” stripped and non-stripped anatomical images were then submitted to New Segment normalization using the default parameters as provided by the authors of the toolbox (SPM manual, FIL Group). Again, this “cleaning” of the anatomical images was only conducted for the lesioned brain group.

This estimation procedure yielded six sets of transformation parameters corresponding to skulled and deskulled data sets submitted to either standard normalization, Unified Segmentation or New Segment. In all cases these transformation parameters were applied to the structural images, the lesion mask, and the functional data if appropriate, i.e. transformation parameters obtained from the New Segment approach were applied to the original, not “cleaned” structural images.

### 2.4 Analysis of structural data

#### 2.4.1 Evaluation of intensity differences between normalized and template images

To assess the general quality of the different normalizations the mean square error (MSE) of intensities was calculated between averaged volumes (mean intensities across all patients) and the brain extracted MNI152 template provided by FSL (Hellier et al., 2003) which we used as the template for normalization in this study. Previous literature has shown that MSE values are useful as comprehensive indicator of general normalization quality and provide a robust statistical measure of intensity similarities (c.f. Razlighi et al. (2013); Ripollés et al. (2012)). The value of MSE is always positive, and is defined such that zero represents the ideal but practically unlikely gold standard of identical image intensities. Since this measure assumes identical MR scanner calibration, all image intensities were scaled to a maximum of one. The averaged brain volume across all patients (one for each of the 4 normalizations), was calculated as the weighted mean for each voxel excluding the individual lesioned brain areas as defined by the lesion mask after normalization for the three lesioned brain groups. Subsequently, the MSE was calculated as the mean squared difference between this weighted averaged image and the reference separately for each normalization using only voxels belonging to the brain of the reference image. That is, voxels belonging to the skull, for example, were left out.

#### 2.4.2 Evaluation of structural differences between normalized and template images

To assess the quality of the various normalization approaches in more detail, differences between normalized brains and the MNI152 template were assessed using a second approach — the DICE Similarity Index (DSI; Dice, 1945). This index measures the overlap between template and individual normalized brain, separately for whole brain, gray matter (GM) and white matter (WM). This index indicates how well the group of normalized images fits to the template and is within the range 0 (no overlap) to 1 (perfect agreement), meaning perfect alignment or similarity. This measure has also repeatedly been used to quantify normalization quality (e.g. Klein et al. (2009); Ripollés et al. (2012)). To this end, the normalized anatomical images resulting from each pipeline as well as the brain extracted MNI152 template were segmented using the “New Segment” approach implemented in SPM8 (SPM manual, FIL Group). The rationale for re-segmentation was the fact that only 2 of the 3 normalizations (Unified Segmentation and New Segment) provide segmented tissue maps. These were generated prior to normalization. In order to avoid bias in further analysis towards one or the other approach, we decided to run a segmentation at this point for all normalization routes, not only for standard normalization. The DSI was then calculated for the whole brain as well as for WM and GM for each normalization separately using the segmented MNI152 template as a reference. Results were compared using random effect analyses of variance (RFX-ANOVA) with the within subject factors Skull (skulled/deskulled) and Normalization (standard/unified/new segment) and the between subject factor Group (no-lesion, small-lesion, medium-lesion, large-lesion). These ANOVAs were calculated separately first for the whole brain, disregarding tissue types, and then for the two tissue types of interest (gray matter, white matter) resulting from the re-segmentation.

Finally, a visual inspection of all brains was performed by two of the authors (RB, FF) evaluating every patients' normalized brain from all 6 pipelines with a focus on the pipelines with the largest DICE difference (see Fig. 5). This was carried out to identify poor normalization and segmentation results and to ensure that DICE values (see below) corresponded to visible outcomes.

### 2.5 Analysis of functional data

Following normalization using the six pipelines, all functional images were spatially smoothed using a Gaussian kernel (FWHM = 5 mm). For single subject analysis, statistical parametric maps were calculated separately for each run using a General Linear Model that included a single regressor representing the activity phase, convolved with a canonical hemodynamic response function. Six nuisance regressors, corresponding to the motion realignment parameters were also included in the model to regress out residual motion artifacts. For this single subject analysis standard default parameters were used, i.e. the model included a high-pass filter of 128 s as well as an AR(1) term. The resulting statistical maps for the regressor of interest were combined across all runs to form one contrast image representing language-related activations.

In order to address activation differences between the different normalization pipelines a random effects repeated measures 2 × 3 × 4 ANOVA was calculated with the within subject factors Skull (skulled/deskulled) and Normalization (standard normalization, Unified Segmentation, New Segment) and the between subject factor Group (no-lesion, small-lesion, medium-lesion, large-lesion). For the calculation of this model a repeated measures GLM with partitioned error variances (in which between-subject and within-subject error terms are modeled separately) was used, allowing between-subject and within-subject effects to be tested within one model.

Statistical parametric maps were thresholded using a voxel-wise p < 0.001. Since our primary interest was in clinically relevant effects, all data were masked exclusively for an extended temporoparietal ROI (including Wernicke's area) and an extended inferior frontal ROI (including Broca's area) using automated anatomical labeling (AAL; Tzourio-Mazoyer et al., 2002) and the Wake Forest University PickAtlas (WFU; Maldjian et al., 2003). In addition, an individual neuroanatomical assessment of functional localization was performed. Statistical t-maps were overlaid onto the warped individual anatomical image and onto the MNI152 template for visual inspection of functional activation after normalization. The relative position of primary functional clusters (Wernicke and Broca) and ROI peak activation (peak t-value) in relation to individual neuroanatomy was evaluated by two of the authors (RB, FF, see Fig. 8). For this, the patients' independent (non-normalized) clinical fMRI results, which are used in pre-surgical planning (Beisteiner et al., 2000) and which have been verified via intraoperative cortical stimulation (see Roessler et al. (2005b)), served as a gold standard.

To check whether brain activation changes more when the lesion is closer to activation, we tested effects of “lesion-to-activation-distance” on normalization differences within the Wernicke area. For this we calculated the Euclidian distance between the lesion (border of the lesion mask) and the peak activation for every patient on original non-transformed functional EPI data. This generated the “lesion-to-activation-distance”. Since the main focus of our study was on differences between skulled/deskulled input data, we then checked the influence of “lesion-to-activation-distance” on “differences in peak voxel location” between skulled and deskulled data by calculating corresponding correlations for all 3 normalizations.

Based on the hypothesis that within subject differences in normalization quality will also lead to differences in the MNI localization of the peak activation, we correlated the maximum DICE difference (gray matter) and the corresponding Euclidian distance of peak activations and tested whether the resulting Pearson's r was positive and significantly different from zero. To this end, we quantified the largest from all pairwise DICE differences per participant and calculated the Euclidian distance between the peak activations within Wernicke area of those two corresponding normalization pipelines.

## 3 Results

### 3.1 Structural analysis

#### 3.1.1 Structural T1 differences within postprocessing Step I

There was very good congruence between skulled and deskulled T1 images after registration to EPI and the entirety of Step I (all DICE coefficients > 0.98 for all analyses and lesion groups). This indicates that the structural differences described below are introduced during *normalization* (Step II) of the skulled/deskulled images. DICE results were also confirmed via subject-wise visual inspection of overlaid images (skulled overlaid on deskulled).

#### 3.1.2 Evaluation of intensity differences between normalized and template images

The mean squared error in intensities revealed a general improvement in the quality of the normalization for skull-stripping (for details, see Table 2). The mean MSE value for deskulled images was 0.13 and the mean MSE for skulled images was 0.19. In addition, the New Segment normalization clearly outperformed the 2 other normalization techniques. The size of brain lesions also affected results. Normalization quality was worse with larger brain lesions. For no-lesion/small-lesion the mean MSE value was 0.12, for medium-lesion/large-lesion it was 0.21.

#### 3.1.3 Evaluation of structural differences between normalized and template images

**Fig. 4:**
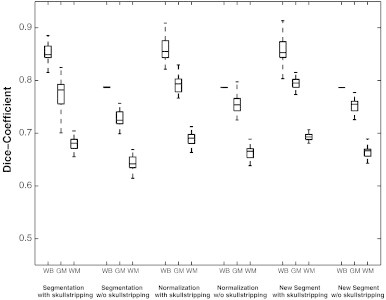
DICE-values for the different normalization pipelines, for whole-brain (WB), gray-matter segmentation (GM) and white-matter segmentation (WM) separately for the six normalization pipelines and across the four lesion size groups. For detailed values separated by lesion size see Tables 3a and 3b.

**Fig. 5:**
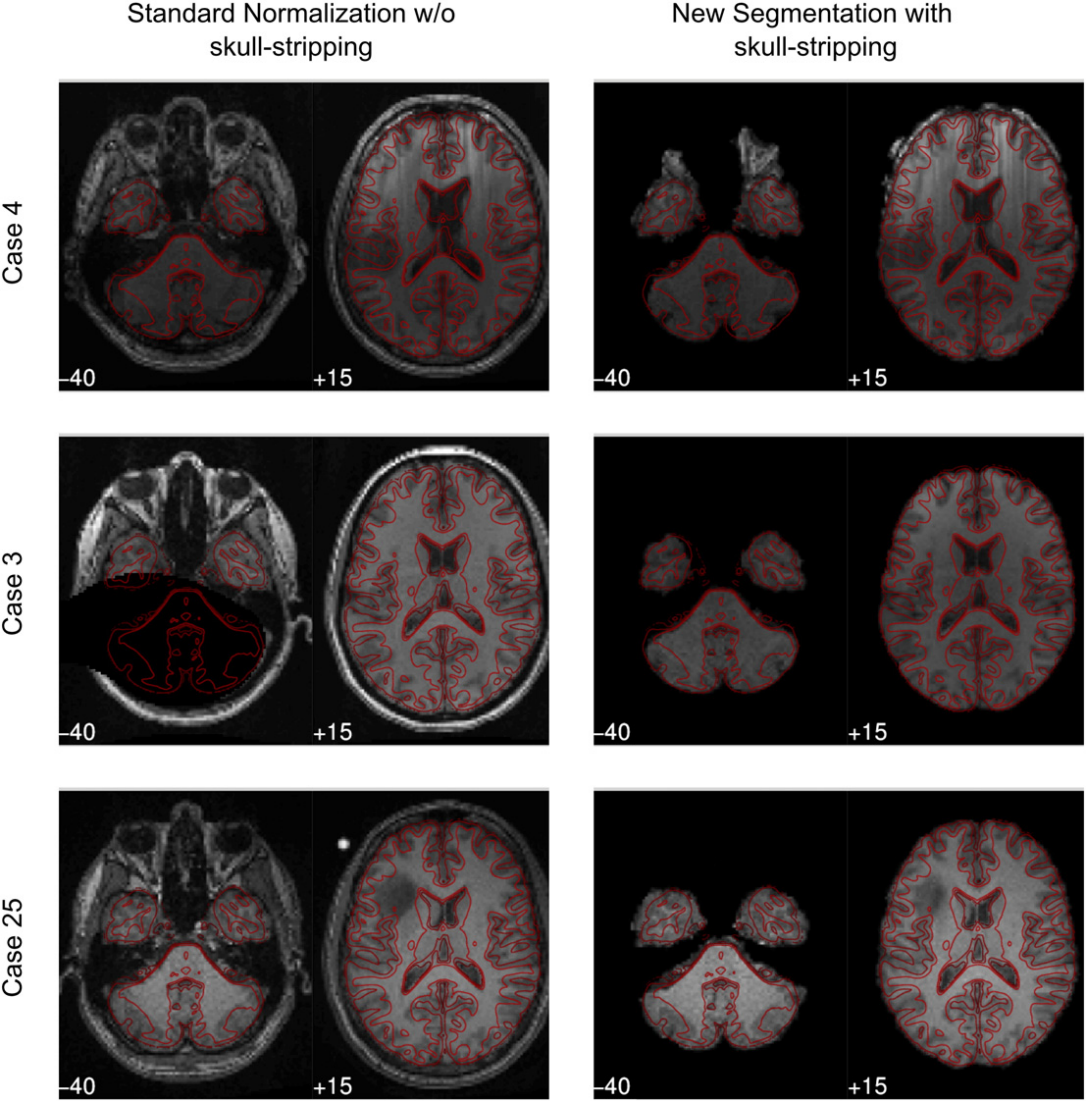
Examples for misaligned brains. Patients with a large (top and middle row, cases 4 and 3) or a small (bottom row, case 25) difference in DICE indices. Most of the patients showed the largest DICE difference between standard normalization without skull-stripping and New Segment with skull-stripping. MNI slices z: − 40 and z: + 15 are shown. The MNI template is outlined in red. Note the considerable mismatch within ventricular planes (+ 15) in the top row and the mismatch within basal planes (− 40) for case 3. Case 25 (bottom row) with similar DICE values for all 6 pipelines shows also similar brain alignments. (For interpretation of the references to color in this figure legend, the reader is referred to the web version of this article.)

DICE coefficients used to assess the quality of the different approaches were submitted to ANOVAs. Detailed results are shown in Tables 3a and 3b and depicted in Figs. 4 and 5. The dominant finding was a significant improvement of template congruence for the skull-stripped images in every tissue category (whole brain, gray matter, white matter). Further parts of the analysis (main effects and interactions) indicated that template congruence was worse with older normalization techniques and larger brain lesions. All findings could be confirmed by the visual qualitative control (see Fig. 5).

### 3.2 Functional analysis

**Fig. 6:**
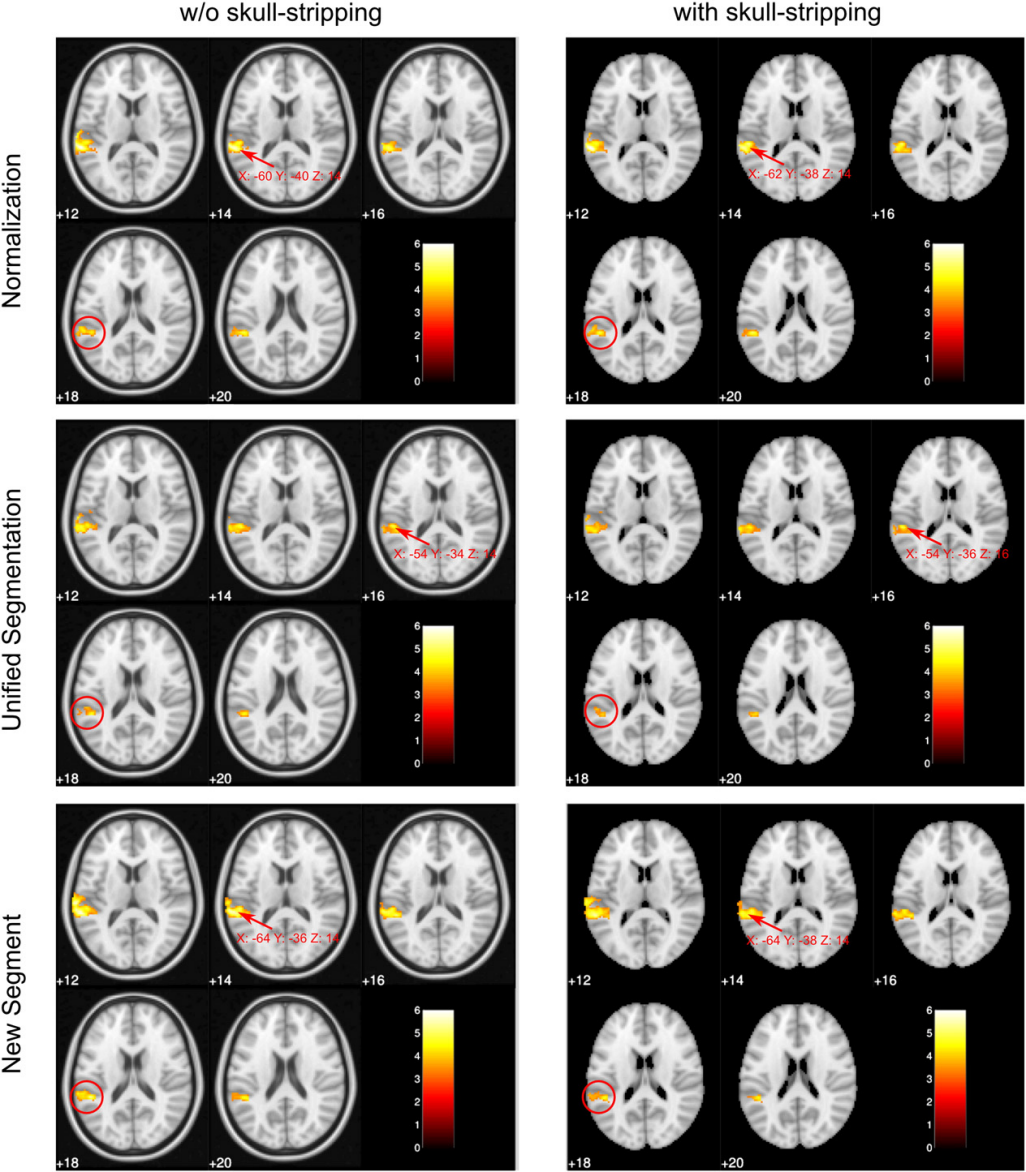
One-sample t-test group results. Significant activation above a threshold of p < 0.001 uncorrected is overlaid on the brain extracted or the standard MNI152 templates provided by FSL. Note that the position of the activation cluster differs (c.f. slice 18 showing almost no activation for the Unified Segmentation Model with skull-stripping as indicated with a red circle) and the Wernicke peak-voxel is shifted between normalization pipelines > 1 cm (indicated with an arrow, locations are given in MNI coordinates). Only slices covering the Wernicke area are shown. (For interpretation of the references to color in this figure legend, the reader is referred to the web version of this article.)

**Fig. 7:**
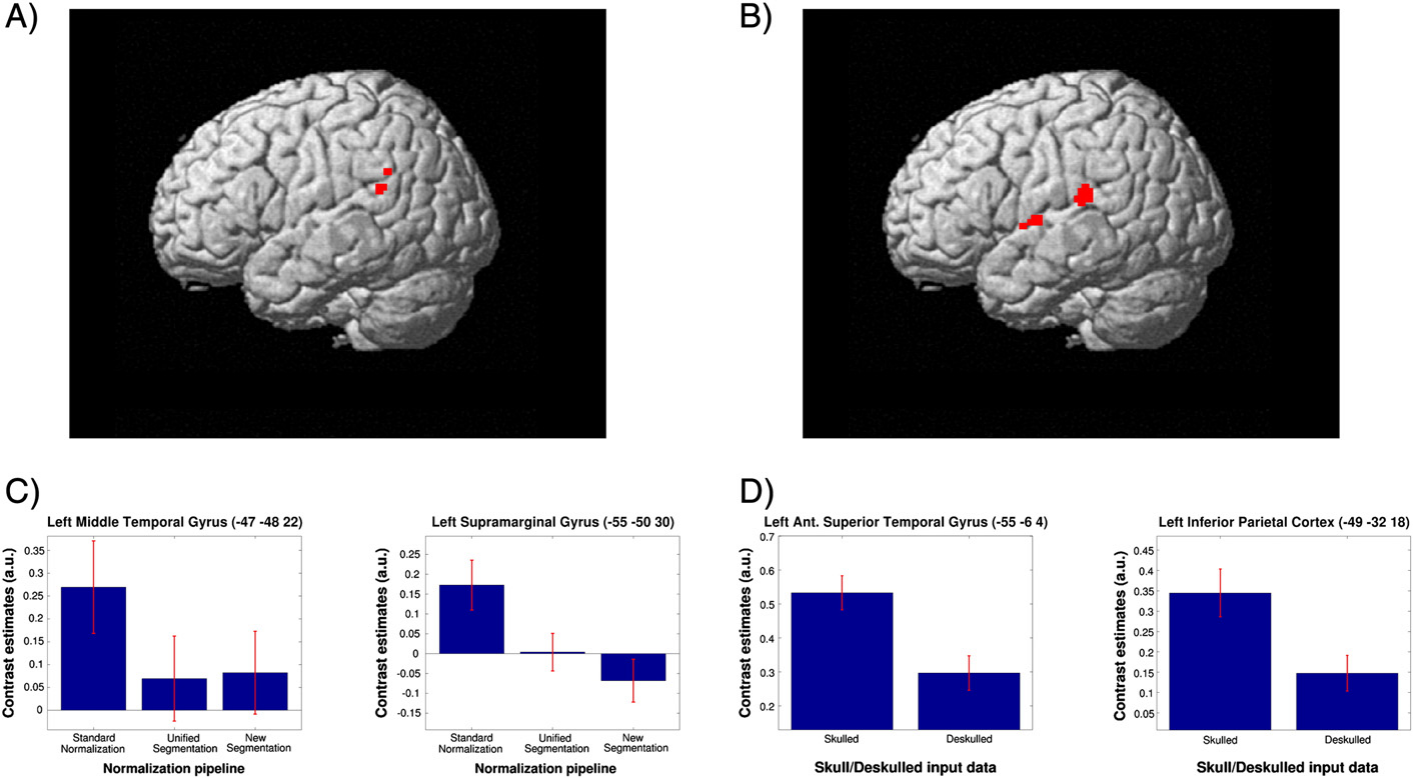
Main effects and contrasts of the 2 × 3 × 4 RFX-ANOVA: Activation differences found for the two main effects “normalization” (in left supramarginal gyrus and left middle temporal gyrus) (A) and “skull-stripping” (in left anterior superior temporal gyrus and left inferior parietal cortex) (B) are shown, rendered onto the SPM5 single-subject brain template. Contrast estimates for all significant brain areas are shown in panels C and D. Anatomical regions with MNI-coordinates and location of the peak-voxel within each cluster can be found in Table 4. All data are masked exclusively for Wernicke's area.

**Fig. 8:**
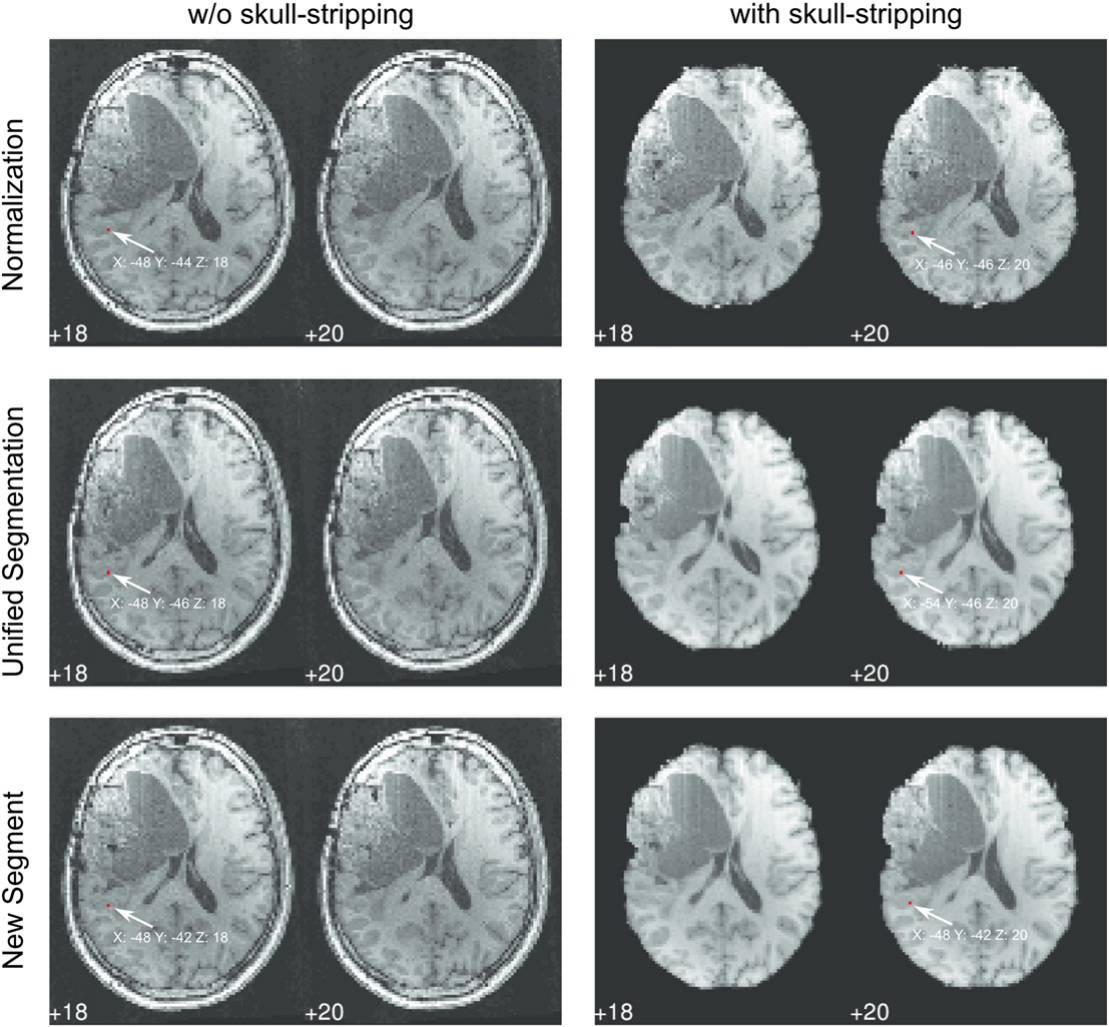
Brain position and MNI coordinates of peak voxel location for a representative patient (case 6) resulting from the 6 normalization pipelines. Note that the Wernicke peak-voxel is located in the same neuroanatomical region, yet this region is shifted in the MNI space.

Reliable task related activations were found within Wernicke and Broca AAL regions and other brain areas as described previously (Foki et al., 2008; Gartus et al., 2009). Detailed results of the 2 × 3 × 4 RFX-ANOVA are shown in Table 4 and illustrated in Figs. 6–8. Concerning general pipeline dependent localization effects, the 6 different normalization pipelines shifted the Wernicke peak more than 1 cm within the MNI space (group data, Fig. 7). The ANOVA generated 3 significant results: a main effect Skull, a main effect Normalization and an interaction Skull × Group. Skull stripping specifically affected the cortex adjacent to Wernicke's core area, which is located in the posterior superior temporal gyrus. Skulled input data showed larger activations in inferior parietal cortex and in the anterior superior temporal gyrus — both outside of the classical Wernicke core (Fig. 7B, D). Analysis of the significant Skull × Group effect (again in inferior parietal cortex) indicated that the skulled > deskulled differences are primarily driven by the medium and large lesion groups.

The normalization techniques affected functional results in a similar way, generating significant differences adjacent to the Wernicke core. While Unified Segmentation and New Segment showed comparable functional signals, standard normalization generated much larger activation in inferior parietal cortex (supramarginal gyrus) and middle temporal gyrus — again both outside of the classical Wernicke core (Fig. 7A, C).

Concerning the question of whether Wernicke activation changes more when the lesion is closer to activation, no significant correlation was found. The shift of peak activation between skulled and deskulled brains did not correlate with individual lesion-to-activation-distances (Wernicke ROI: Standard Normalization: r = − 0.24, Unified Segmentation: r = − 0.16, New Segment: r = 0.1). However, our hypothesis is that differences in DICE indices reflect localization differences of the peak activation (r = 0.28, p = 0.0402). This indicates that an increase in the deviation between brain and template also increases the shift of functional activations in MNI space.

### 3.3 Neuroanatomical assessment of individual fMRI activations

The changes in MNI coordinates of group activation clusters (Fig. 7) were further elucidated by a qualitative single subject analysis (Fig. 8) in which locations were compared with those established in the clinical patient reports (Beisteiner et al., 2000, 2008; Roessler et al., 2005a). This revealed that activation strength, cluster size and position of activation clusters relative to surrounding neuroanatomy were quite stable. In addition, the atypical “Wernicke activations” found for not-deskulled input data and standard normalization (inferior parietal, anterior superior temporal, middle temporal) were not evident in the patient reports based on standard clinical thresholds. A secondary analysis of the group data (the details of which we do not report) confirmed these individual qualitative findings by demonstrating that group cluster sizes and group cluster t-values did not differ between the 6 normalization pipelines. However, as is also evident from the visual analysis of the DICE index differences (Fig. 5), the overall brain positions varied in MNI space depending on the normalization pipeline. The consequence of this finding is that the MNI coordinates of an activation cluster change despite keeping a rather stable position within the individual brain (Fig. 8). Correspondingly, MNI peak activation coordinates varied depending on the normalization pipeline — typically below 1 cm, but up to 4.6 cm with one outlier patient.

## 4 Discussion

Our study provides 2 major results: (1) structural analysis indicates that the most reliable MNI coordinates are achieved using *deskulled input data* — particularly when combined with new normalization techniques (Unified Segmentation, New Segment). (2) As a consequence, the MNI coordinates of essential language activations may be partly misleading with skulled input data sets — particularly when combined with standard normalization. This specifically concerns parietal and temporal cortex.

In more detail, we found that the skulled brains, for which normalization quality was inferior (see Fig. 5) lead to misleading activation. This is shown by a significant skulled > deskulled activation in the left inferior parietal cortex (− 49 − 32 18 in Table 4, red circles in Fig. 6) and left anterior superior temporal gyrus (− 55 − 6 4 in Table 4) — both clearly outside the classical Wernicke core. The term “misleading” seems justified for 3 reasons: (1) the remote parietal and temporal activations were not seen with standard clinical thresholds (clinical patient reports), (2) the larger the mismatch between template and brain (which was largest with skulled data), the greater the change in the location of Wernicke activation, and (3) no pipeline changed Wernicke activations significantly in relation to local neuroanatomy (Fig. 8), but neuroanatomy changed in relation to the MNI coordinates (i.e. a brain–template mismatch occurred with skulled data). Therefore, the conclusion must be that normalization of skulled brains shifts part of the “correct” Wernicke activations to “wrong” MNI coordinates in the temporo-parietal cortex. A similar temporo-parietal effect was found for the standard normalization technique. Standard normalization generated a misleading activation increase in left supramarginal gyrus and left middle temporal gyrus outside the Wernicke core.

Details of the structural analysis revealed that the most important factor for improvement of the congruence between the MNI template and normalized brains (MSE values, DICE coefficients, Figs. 4 and 5) was skull-stripping. Further, the new normalization techniques outperformed standard normalization with New Segment proving to be the best approach. Evaluation of the procedures required for generation of a uniform starting-point of skulled/deskulled brains (Step I) indicated that the decisive differences between the 6 normalization pipelines were introduced during Step II.

With regard to lesion size effects, a systematic influence was found for the structural data: brains with larger lesions differed more significantly from the template than brains with smaller lesions. With the functional data, lesion size tended to increase the functional mislocalizations (larger parietal skulled > deskulled effects). The distance between brain lesion and brain activation however, did not significantly affect normalization quality.

Summarizing our functional and structural findings (Figs. 5–8), the primary cause of our activation differences is a differing quality of alignment between normalized brains and MNI template. This leads to shifts in activation clusters and peak activations (maximum 4.6 cm) within MNI space. Skull-stripping the input data is the most important factor in improving this. Clearly, the implicit skull-stripping step, already implemented in most normalization algorithms, does not produce results of the same quality as explicitly editing the input data. With standard normalization, implicit skull-stripping is realized by weighting of non-brain voxels to exclude non-brain structures after an initial affine transformation but prior to nonlinear normalization. With Unified Segmentation (Ashburner and Friston, 2005) tissue probability maps for gray matter, white matter, cerebrospinal fluid (CSF) and a fourth map for the residuals are generated. The latter implicitly accounts for the skull and the scalp. However, in concordance with Fein et al. (2006) and Acosta-Cabronero et al.(2008) we found a clear benefit for normalization quality if deskulled data are used as primary input for the normalization process.

Interestingly, the left temporoparietal position of our functional differences corresponds to the left temporal differences found for different normalization algorithms in the work of Crinion et al. (2007), who also investigated language data but not skull-stripping effects. Their and our results indicate that temporal areas are a specific source of structural variability during the normalization process with current templates. Besides choosing optimized postprocessing techniques it seems sensible to recommend that group studies, where critical activations are expected in temporal brain areas, include a series of single-patient analyses to check for internal consistency of the structural and functional data. A further issue of special clinical relevance concerns activations in other brain areas comprising essential cortex. MNI peak activation shifts of the size found here (> 1 cm with the group data, > 4 cm individually) may easily become critical. For example in primary sensorimotor cortex around the central sulcus, such a peak activation shift within MNI space may well decide between concluding that the main result of a study is primary motor activation or a primary sensory activation. Therefore, skull-stripping of the input data should become standard, but not only for clinical studies. It can be performed either as a separate step or by inclusion in standard analysis pipelines, as already suggested for non-human data (Budin et al., 2013). Our implementation of skull-stripping with the BET2 software requires considerable manual postprocessing to obtain optimal removal of non-brain areas. Newer and potentially more accurate algorithms, such as the simplex mesh and histogram algorithm (SMHASS; Galdames et al., 2012), may be candidates for integration into fully automated routines.

In conclusion, we have shown that combining deskulled input data with the New Segment normalization technique generates the highest probability of achieving valid MNI coordinates for functional activations. The functional and structural variability described is relevant for functional conclusions in a clinical context and should also be considered when comparing MNI coordinates from different fMRI studies.

## Figures and Tables

**Fig. 2:**
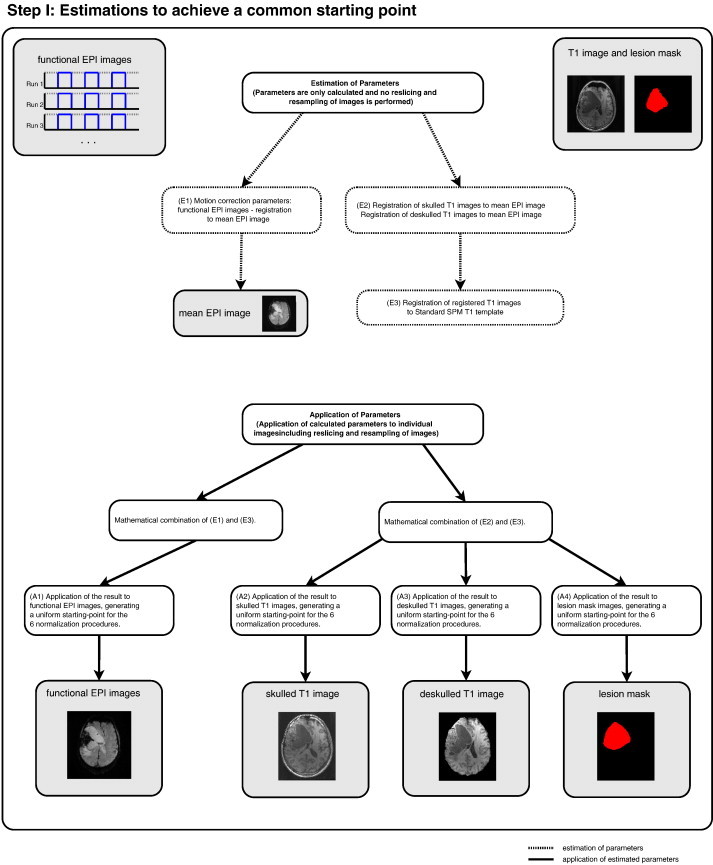
Flow chart delineating the preprocessing steps undertaken to achieve a common starting point for the 6 normalization pipelines, i.e. estimation steps to transform the data into MNI space. See text for further details.

**Fig. 3:**
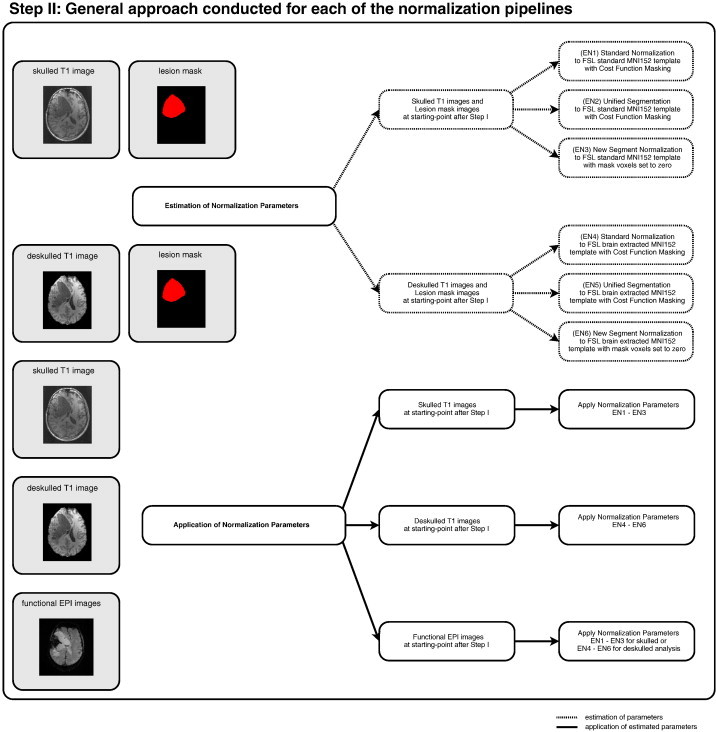
Flow chart delineating the normalization steps illustrating the general approach conducted for each of the six normalization pipelines. See text for further details.

**Table 1:** Patient characteristics including sex, age, diagnosis and lesion size in cm^3^. Note that cases 31 to 36 were classified as controls since clinical evaluation showed no structural or functional finding except for epilepsy.

Case number	Sex	Age	Diagnosis	Lesion size (cm^3^)
Case 1	Female	45	Low grade glioma, left temporal	44.72
Case 2	Male	43	Tumor of unknown origin, left postcentral	236.29
Case 3	Male	32	Oligodendroglioma, left insular cortex	253.21
Case 4	Male	68	Tumor of unknown origin, left postcentral	65.12
Case 5	Male	34	Astrocytoma grade II, left fronto-temporal	516.69
Case 6	Male	45	Astrocytoma, left frontal-temporal	564.20
Case 7	Male	50	Glioma grade II, left temporal cortex	504.90
Case 8	Female	40	Astrocytoma, left temporoparietal	540.22
Case 9	Male	51	Glioma, left frontal cortex	201.71
Case 10	Male	38	Astrocytoma grade II, left temporal cortex	195.32
Case 11	Female	33	Astrocytoma grade II, left frontotemporal	24.82
Case 12	Female	54	Tumor of unknown origin, left parietal	147.24
Case 13	Female	30	Cavernous hemangioma, left frontal	2.28
Case 14	Male	65	Astrocytoma grade II, left opercular cortex	102.01
Case 15	Female	27	Tumor of unknown origin, left temporal cortex	173.55
Case 16	Female	37	Oligoastrocytoma grade II, left opercular	271.08
Case 17	Female	49	Glioma grade III, left temporoparietal	46.73
Case 18	Male	37	Low grade glioma, left temporal cortex	414.60
Case 19	Male	69	Tumor of unknown origin, left temporo-parietal cortex	209.93
Case 20	Male	38	Low grade glioma, left frontal	7.186
Case 21	Male	52	Tumor of unknown origin, left frontal	274.19
Case 22	Male	34	Cavernous hemangioma, left basal ganglia	36.74
Case 23	Male	21	Astrocytoma, left postcentral	5.02
Case 24	Male	37	Tumor of unknown origin, left frontotemporal	49.58
Case 25	Female	60	Tumor of unknown origin, left fronto-central	148.98
Case 26	Male	45	Tumor of unknown origin, left frontal	11.75
Case 27	Female	75	Tumor of unknown origin, left temporal cortex	296.98
Case 28	Male	45	Tumor of unknown origin, left fronto-temporal	400.59
Case 29	Female	55	Tumor of unknown origin, left precentral	51.56
Case 30	Male	33	Low grade glioma, left insular cortex	96.15
Case 31	Female	19	Temporal lobe epilepsy left	–
Case 32	Male	20	Temporal lobe epilepsy left	–
Case 33	Male	21	Temporal lobe epilepsy left	–
Case 34	Female	47	Temporal lobe epilepsy left	–
Case 35	Male	49	Temporal lobe epilepsy left	–
Case 36	Female	32	Temporal lobe epilepsy left	–
Case 37	Male	35	Healthy participant	–
Case 38	Female	43	Healthy participant	–
Case 39	Female	30	Healthy participant	–
Case 40	Male	27	Healthy participant	–

**Table 2:** MSE coefficients for the different normalization pipelines separately for the four different lesion size groups (no-lesion, small-lesion, medium-lesion, large-lesion).

	No-lesion	Small-lesion	Medium-lesion	Large-lesion
Skulled brains: Standard Normalization	0.1003	0.3044	0.2999	0.2892
Deskulled brains: Standard Normalization	0.0462	0.2108	0.2408	0.2302
Skulled brains: Unified Segmentation	0.1032	0.1881	0.3113	0.2625
Deskulled brains: Unified Segmentation	0.0360	0.1428	0.2800	0.2253
Skulled brains: New Segment	0.1068	0.1120	0.1173	0.1304
Deskulled brains: New Segment	0.0332	0.0373	0.0276	0.0656

**Table 3a:** Mean DICE coefficients and results from the repeated-measures ANOVA using the factors Group (no-lesion, small-lesion, medium-lesion, and large-lesion), Normalization (Standard Normalization, Unified Segmentation, New Segmentation) and Skull (deskulled, skulled images) separately for whole-brain, gray-matter and white matter. Only significant effects with their corresponding mean DICE coefficients (standard error is given in brackets) are given.

Whole-brain analysis:
Main effect Skull: F = 1141.209; df = 1,36; p < 0.000
Deskulled: 0.857 (0.002); skulled: 0.786 (0.002)
Main effect Group: F = 5.258; df = 3,36; p < 0.004
No-lesion: 0.830 (0.003); small: 0.820 (0.003); medium: 0.819 (0.003); large: 0.817 (0.003)
Interaction effect Skull × Group: F = 8.590; df = 3,36; p < 0.000
Deskulled: No-lesion: 0.875 (0.005); small: 0.853 (0.005); medium: 0.851 (0.005); large: 0.849 (0.005) Skulled: No-lesion: 0.786 (0.001); small: 0.788 (0.001); medium: 0.787 (0.001); large: 0.785 (0.001)

Gray-matter analysis:
Main effect Skull: F = 127.169; df = 1,36; p < 0.000
Deskulled: 0.857 (0.002); skulled: 0.786 (0.002)
Main effect Normalization: F = 7.207; df = 2,72; p < 0.001
Normalization: 0.747 (0.004); Unified Segmentation: 0.764 (0.008); New Segment: 0.779 (0.002)
Interaction effect Normalization × Skull: F = 7.859; df = 2,72; p < 0.001
Deskulled: Normalization: 0.771 (0.005); Unified Segmentation: 0.781 (0.09); New Segmentation: 0.788 (0.004) Skulled: Normalization: 0.723 (0.004); Unified Segmentation: 0.746 (0.007); New Segmentation: 0.752 (0.002)
Interaction effect Normalization × Skull × Group: F = 3.387; df = 6,36; p > 0.005
See Table 3b for details on mean DICE coefficients.

White-matter analysis:
Main effect Skull: F = 234.189; df = 1,36; p < 0.000
Deskulled: 0.682 (0.003); skulled: 0.653 (0.003)
Interaction Normalization × Skull: F = 14.312; df = 2,72; p < 0.000
Deskulled: Normalization: 0.679 (0.002); Unified Segmentation: 0.679 (0.007); New Segmentation: 0.687 (0.004) Skulled: Normalization: 0.642 (0.003); Unified Segmentation: 0.656 (0.006); New Segmentation: 0.661 (0.003)

**Table 3b:** DICE coefficients for the different normalization pipelines separately for whole-brain (WB), gray-matter segmentation (GM) and white-matter segmentation (WM) (see also Fig. 4).

Structure	Deskulled brains: Normalization	Skulled brains: Normalization	Deskulled brains: Unified Segmentation	Skulled brains: Unified Segmentation	Deskulled brains: New Segment	Skulled brains: New Segment
*No-lesion Group:*						
Whole brain analysis	0.8659	0.7825	0.8690	0.7881	0.8898	0.7870
Gray-matter segmentation	0.7371	0.7072	0.7832	0.7432	0.7887	0.7597
White-matter segmentation	0.6809	0.6453	0.6716	0.6501	0.6924	0.6719

*Small-lesion Group:*						
Whole brain analysis	0.8506	0.7889	0.8551	0.7869	0.8528	0.7868
Gray-matter segmentation	0.7776	0.7269	0.7784	0.7373	0.7984	0.7507
White-matter segmentation	0.6845	0.6515	0.6813	0.6570	0.6967	0.6679

*Medium-lesion Group:*						
Whole brain analysis	0.8513	0.7868	0.8519	0.7870	0.8499	0.7869
Gray-matter segmentation	0.7968	0.7284	0.7907	0.7541	0.7940	0.7502
White-matter segmentation	0.6808	0.6364	0.6884	0.6617	0.6926	0.6617

*Large-lesion Group:*						
Whole brain analysis	0.8447	0.7843	0.8560	0.7873	0.8454	0.7822
Gray-matter segmentation	0.7706	0.7281	0.7735	0.7509	0.7712	0.7476
White-matter segmentation	0.6699	0.6334	0.6761	0.6550	0.6681	0.6431

**Table 4:** Results of the 2 × 3 × 4 RFX-ANOVA (p = 0.001 uncorr): Anatomical regions with MNI-coordinates and location of the peak-voxel within each cluster are given.

Anatomical region — location (area)	x, y, z (mm)	F
**Main effect skull:**		
**Left anterior superior temporal gyrus**	− 55 − 6 4	23.746
**Left inferior parietal cortex**	− 49 − 32 18	19.80
**Main effect normalization:**		
**Left middle temporal gyrus**	− 47 − 48 22	14.179
**Left supramarginal gyrus**	− 55 − 50 30	10.961
**Interaction effect Skull** × **Group**		
**Left supramarginal gyrus**	− 51 − 50 26	8.091

Note that all activations listed are significant at p < 0.001 uncorrected. Per cluster center (bold face) maximal 2 additional local maxima were listed > 8.0 mm apart.
